# Phosphoproteome Analysis of Invasion and Metastasis-Related Factors in Pancreatic Cancer Cells

**DOI:** 10.1371/journal.pone.0152280

**Published:** 2016-03-25

**Authors:** Xiaodong Tan, Peng Liu, Yinpeng Huang, Lei Zhou, Yifan Yang, Huaitao Wang, Boqiang Yu, Xiangli Meng, Xiaobo Zhang, Feng Gao

**Affiliations:** Department of General Surgery, Shengjing Hospital of China Medical University, Shenyang, Liaoning, P.R.China; Harbin Medical University, CHINA

## Abstract

Mechanisms of abnormal protein phosphorylation that regulate cell invasion and metastasis in pancreatic cancer remain obscure. In this study, we used high-throughput phosphorylation array to test two pancreatic cancer cell lines (PC-1 cells with a low, and PC-1.0 cells with a high potential for invasion and metastasis). We noted that a total of 57 proteins revealed a differential expression (fold change ≥ 2.0). Six candidate proteins were further validated by western blot with results found to be accordance with the array. Of 57 proteins, 32 up-regulated proteins (e.g. CaMK1-α and P90RSK) were mainly involved in ErbB and neurotrophin signaling pathways as determined using DAVID software, while 25 down-regulated proteins (e.g. BID and BRCA1) were closely involved in apoptosis and p53 signaling pathways. Moreover, four proteins (AKT1, Chk2, p53 and P70S6K) with different phosphorylation sites were found, not only among up-regulated, but also among down-regulated proteins. Importantly, specific phosphorylation sites can affect cell biological functions. CentiScaPe software calculated topological characteristics of each node in the protein-protein interaction (PPI) network: we found that AKT1 owns the maximum node degrees and betweenness in the up-regulation protein PPI network (26 nodes, average path length: 1.89, node degrees: 6.62±4.18, betweenness: 22.23±35.72), and p53 in the down-regulation protein PPI network (17 nodes, average path length: 2.04, node degrees: 3.65±2.47, betweenness: 16.59±29.58). In conclusion, the identification of abnormal protein phosphorylation related to invasion and metastasis may allow us to identify new biomarkers in an effort to develop novel therapeutic drug targets for pancreatic cancer treatment.

## Introduction

Pancreatic cancer is a highly malignant disease with a very poor prognosis. Despite considerable advances in radiological and endoscopic ultrasound techniques, it often presents as a locally advanced or metastatic disease in most patients, and only about 10–20% of patients are considered candidates to surgery [1, 2]. Apart from surgery, other effective methods of treatment do not exist, and the survival rate for resected patients is also extremely low. The major reason for a poor prognosis is local recurrences and/or distant metastasis after surgery. This suggests that understanding the cellular and molecular mechanisms involved in invasion and metastasis of pancreatic cancer is important and requires further exploration.

However, protein post-translation modifications in pancreatic cancer cell lines, which may play essential roles in the regulation of cellular responses, have not been clearly demonstrated. It is therefore important to identify any phosphorylation events and to determine whole protein phosphorylation profiles of tumor cells. Recently, by comparing the phosphoproteomes at various developmental stages of skin cancer in mice, proteins associated with early and late cellular responses were identified, providing new insights into the progression of the disease [3]. Batchu et al. found that miR-26a treatment could restore wild-type functions of mutant p53 via phosphorylation at its Ser9 and Ser392 residues, resulting in inhibition of cell growth [4]. Therefore, site-specific phosphorylation of proteins plays an important role in regulating cell processes.

However, as far as we know, studies have not analyzed the phosphoproteome profiles of the two homogenous cell lines (PC-1 with a low, and PC-1.0 with a high potential of invasion and metastasis) [5, 6] in pancreatic cancer research. Our aim is to compare changes on 582 phosphorylation sites of 452 proteins between PC-1 and PC-1.0 cells by utilizing Phospho Explorer Antibody Array technology. In addition, the pathways and networks that related to phosphoproteins identified in our study show important variations in their components.

## Materials and Methods

### Cell lines and cell culture

Two hamster pancreatic cancer cell lines were used: weakly invasive and metastatic cells (PC-1), and highly invasive and metastatic cells (PC-1.0). The PC-1 cell line was established from pancreatic ductal adenocarcinomas induced by BOP in a Syrian golden hamster. The PC-1.0 cell line was established from a subcutaneous tumor produced after inoculation of a hamster by PC-1 cells [5,6]. *In vitro*, PC-1.0 cells are mainly single cells, whereas PC-1 cells grow as island-like cell colonies. *In vivo*, local invasion by PC-1.0 cells and local expansion of PC-1 cells were observed [7].

PC-1.0 and PC-1 cells were incubated in RPMI-1640 (Gibco-BRL, Grand Island, NY, USA), supplemented with 10% fetal bovine serum (Bioserum, Canterbury, Victoria, Australia), 100 U/mL penicillin G and 100 μg/mL streptomycin at 37°C in a humidified atmosphere of 5% CO_2_/95% air.

### Phospho-protein profiling by Phospho Explorer Antibody Array

Cell lysates obtained from PC-1 and PC-1.0 cells were applied to a Phospho Explorer Antibody Array (Full Moon Biosystems, Sunnyvale, CA, USA). The array contained 1318 antibodies. Each of the antibodies has two replicates that are printed on coated glass microscope slide, along with multiple positive and negative controls. Briefly, cell lysates were extracted with Antibody Array Assay Kit that contained a protease inhibitor cocktail and phosphatase inhibitor, and were performed according to the manufacturer's protocol. 25 μg of cell lysates were labeled with 3 μl of biotin. Antibody microarray slides were first blocked in a blocking solution for 30 minutes at room temperature, rinsed with Milli-Q grade water, and dried with compressed nitrogen. And then incubated with biotin-labeled cell lysates in coupling solution at room temperature for 2 h. Array slides were washed four times with 60ml of 1× Wash Solution and rinsed extensively with Milli-Q grade water before detection of bound biotinylated proteins using Cy3-conjugated streptavidin. The slides were scanned on a GenePix 4000 scanner and the images were analyzed with GenePix Pro 6.0 (Molecular Devices, Sunnyvale, CA, USA).

A phosphorylation ratio change was computed based on the following equation where expression of phosphorylated proteins was normalized to corresponding unphosphorylated protein expression in both experimental (PC-1.0) and reference data (PC-1). Phosphorylated proteins were considered to be differentially expressed when an increase (≥ 2.0) or decrease (≤ 0.5) occurred in the ratio of expression levels between PC-1.0 and PC-1 cells. Protein phosphorylation data were confirmed by western blot.

### Antibodies And Reagents

Rabbit polyclonal antibodies raised against epitopes of human Abl1, pAbl1-Tyr204 (Cell Signaling Technology, Danvers, MA, USA), ITGB4, ITGB4-Tyr1510 (Abcam, Cambridge, MA, USA), Myc, pMyc Ser62, Fos, pFos-Thr232, IRS-1, pIRS-1-Ser636, Raf1, pRaf1-Tyr341 and ß-actin (Santa Cruz Biotechnology, Dallas, TX, USA) were used as primary antibodies. Horseradish peroxidase-conjugated secondary antibodies or FITC-labeled fluorescent antibodies (Santa Cruz Biotechnology, Dallas, TX, USA) were used as secondary antibodies for western blotting. FOS and IRS-1 siRNA were purchased from Santa Cruz Biotechnology (Dallas, TX, USA). RAF1 inhibitor (GW5074) was purchased from Selleck Chemicals (Houston, TX).

### Western blot

Western blotting was performed as described previously [7]. Cells were collected on ice using RIPA buffer supplemented with protease inhibitor cocktail and phosphatase inhibitor for 30 mins. In brief, samples of equivalent total protein (20 μg) were run in a 5% polyacrylamide slab gel and transferred to a polyvinylidene fluoride (PVDF) membrane (Bio-Rad, Anaheim, CA, USA). Membranes were incubated with primary antibody, diluted in 0.1% Tween-20/PBS, overnight at 4°C. Blots were then incubated with horseradish peroxidase-conjugated secondary antibody (diluted 1:5000) in 0.1% Tween-20/PBS. Proteins were visualized using enhanced chemiluminescence detection reagents (Santa Cruz Biotechnology).

### *In vitro* invasion and migration assays

PC-1.0 cells were transiently transfected with different siRNAs using Lipofectamine 2000 (Invitrogen, Grand Island, NY), or were suppressed using RAF1 inhibitor. For invasion Transwell assays, the transfected cells (5×10^4^ cells/mL) in serum-free medium were added to the upper chambers, which was purchased from Costar (8 μm pore-size filters, New York, NY) and coated with Matrigel (dilution1:4). The lower chambers were filled with RPMI-1640 containing 10% serum. After 24 h of incubation, the cells remaining in the upper chambers were removed, and the invasive cells in the lower chambers were fixed with 4% paraformaldehyde (Sigma-Aldrich), stained with crystal violet at room temperature, and counted under a microscope. For wound healing migration assay, the transfected cells were seeded onto 6-well plates for 24 h. A 1mm-wide wound was made across the monolayer using the tip of a 200 μL pipette. Then, photos were taken at 0, 6 and 12 h using a microscope. The PC-1.0 cells were performed as above described as a control. Each experiment was performed in duplicates and for three times. For confirming the level of three proteins in the knockdown cell, cells were subjected to real-time PCR and Western blot analysis using specific primers and antibodies, respectively. Primers used for the amplification of FOS and IRS-1 are listed in S1 Table.

### Gene Ontology and Kyoto Encyclopedia of Genes and Genomes (KEGG) pathway analyses and Search Tool For The Retrieval of Interacting Genes/Proteins

For the detection of significantly enriched Gene Ontology (GO) terms, the Cytoscape plugin BINGO was used [8,9]; differentially expressed phosphorylated proteins were automatically assembled to categories of biological process, molecular function or cellular component. GO groups with a corrected *P*-value < 0.01 were denoted for different significance levels. Pathway analyses of differentially expressed phosphorylated proteins was conducted using DAVID (the Database for Annotation, Visualization and Integration Discovery) bioinformatics resources (http://david.abcc.ncifcrf.gov/) [10] bioinformatics and significantly changed signaling pathways were selected based on a *P*-value < 0.01, and FDR (False Discovery Rate) < 10%. Enrichment could therefore be quantitatively measured using a modified Fisher’s exact test. Using a Search Tool for the Retrieval of Interacting Genes/Proteins (STRING) (http://www.string-db.org/) database, we obtained a protein-protein interaction (PPI) network [11]. A PPI network was constructed when a confidence score of more than 0.9 showed that only interactions with a high level of confidence were considered as valid links in the PPI network.

### Topological analysis of PPI network

For a better understanding of the PPI network, CentiScaPe software [12] were used to analyze topological characteristics of the network and to calculate node degree distribution, average path length, topological coefficient and betweenness. We could directly analyze molecular interaction networks using visualized tools, and obtained interesting key proteins. Moreover, a GeneMANIA (http://www.genemania.org/) web server was employed to predict the networks. Such a web server tool made efficient gene function predictions, including co-expression, physical interactions, pathways and predicted networks, that are based on previously reported experimental data [13]. In this report, we employed an automatically selected weighting method and used human as a source species to predict networks.

### Statistics Analysis

Statistical analysis and graphics were undertaken using GraphPad Prism 6.0 (GraphPad Software, San Diego CA). Results are presented as mean ± standard error of mean (SEM). Comparisons of quantitative data were analyzed by Student’s *t* test or ANOVA between two groups (two-tailed; *P*-values < 0.05 were considered to be statistically significant).

## Results

### Differentially expressed protein phosphorylation identified by PEX100 in highly (PC-1.0) and weakly (PC-1) invasive and metastatic pancreatic cells

To identify differentially expressed signaling-associated phosphorylated proteins between highly (PC-1.0) and weakly (PC-1) invasive and metastatic cancer cells, expression levels of phospho-antibody specific proteins in the two pancreatic cancer cell lines were compared. Of the 1318 antibodies analyzed in microarray experiments, a total of 57 proteins showed differential expression using a fold ratio ≥ 2 as the cutoff criterion. Of these 57 proteins, the expression levels of 32 proteins were markedly increased in PC-1.0 as compared with PC-1 cells (Table 1 shows the ten most upregulated proteins). In contrast, the expression levels of 25 proteins were significantly decreased in PC-1.0 as compared with PC-1 cells (Table 2 shows the ten most downregulated proteins). The ratios shown represent expression levels in PC-1.0 cells compared with expression levels in PC-1 cells. CaMK1-α was over-expressed at high levels in PC-1.0 cells whereas Smad1 was down-regulated, revealing a low array intensity. Moreover, four proteins (AKT1, Chk2, p53 and P70S6K) with different phosphorylation sites were found, not only among up-regulated, but also among down-regulated proteins. The actual array chip images were shown in S2 Table and the expression of all proteins were listed in S3 Table.

**Table 1:** The top ten up-regulated genes in highly invasive and metastatic cells (PC-1.0) compared to weakly invasive and metastatic cells (PC-1).

Name	Gene Name	Phosphorylation Site	PC-1 #	PC-1.0#	Ratio*
**CaMK1-α**	calcium/calmodulin-dependent protein kinase I	Thr177	0.41	1.77	4.32
**P90RSK**	Ribosomal protein S6kinase, 90 kDa, polypeptide 1	Thr573	0.53	2.23	4.21
**Lamin A/C**	lamin A/C	Ser392	0.16	0.55	3.44
**DDX5**	DEAD (Asp-Glu-Ala-Asp) box helicase 5	Tyr593	1.30	4.18	3.22
**Abl1**	ABL proto-oncogene 1, non-receptor tyrosine kinase	Tyr204	0.86	2.42	2.81
**Raf1**	Raf-1 proto-oncogene, serine/threonine kinase	Tyr341	0.75	2.01	2.68
**MSK1**	ribosomal protein S6 kinase, 90kDa, polypeptide 5	Ser376	0.86	2.26	2.63
**ITGB4**	integrin, beta 4	Tyr1510	2.16	5.09	2.36
**IRS-1**	insulin receptor substrate 1	Ser636	0.97	2.26	2.33
**PLCG2**	phospholipase C, gamma 2	Tyr753	0.37	0.86	2.32

# phosphorylation ratio = $\frac{\text{phospho}}{\text{unphospho}}$

* Ratio = $\frac{\text{phosphorylation ratio}\;(\text{PC}-1.0)}{\text{phosphorylation ratio}\;(\text{PC}-1)}$

**Table 2:** The top ten down-regulated genes in highly invasive and metastatic cells (PC-1.0) compared with weakly invasive and metastatic cells (PC-1).

Name	Gene Name	Phosphorylation Site	PC-1	PC-1.0	Ratio
**Smad1**	SMAD family member 1	Ser187	2.53	0.67	0.26
**BID**	BH3 interacting domain death agonist	Ser78	1.51	0.46	0.30
**ACTC1**	Actin, alpha cardiac muscle 1	Tyr55	1.48	0.47	0.32
**Smad2**	SMAD family member 1	Ser250	2.29	0.74	0.32
**GRK1**	G protein-coupled receptor kinase 1	Ser21	2.28	0.79	0.35
**AKT1**	v-akt murine thymoma viral oncogene homolog 1	Ser124	1.29	0.46	0.36
**Cytokeratin 8**	keratin 8, type II	Ser431	3.83	1.45	0.38
**c-Raf**	Raf-1 proto-oncogene, serine/threonine kinase	Ser296	1.03	0.40	0.39
**Smad2**	SMAD family member 2	Thr220	1.55	0.61	0.39
**PDGF R alpha**	platelet-derived growth factor receptor, alpha polypeptide	Tyr849	1.19	0.49	0.41

### Validation of phosphorylation proteins by western blot, and differentially proteins play a major role in cell migration, and invasion of PC-1.0 Metastatic

**Fig 1:**
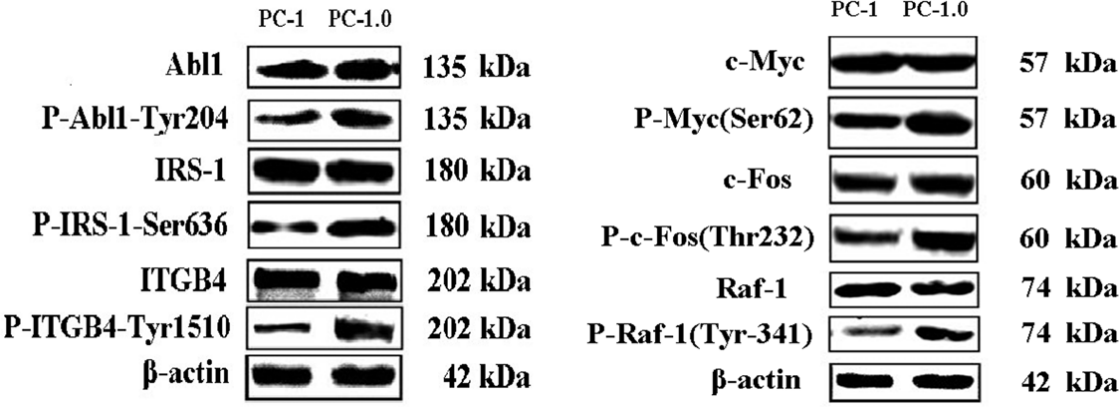
Western blots showing differential phosphorylation of proteins in highly (PC-1.0) and weakly (PC-1) invasive and metastatic pancreatic cancer cells. As shown, the total expression of each protein was equal in both cell lines, while at the phosphorylation level, the expression of six proteins was stronger in PC-1.0 cells than PC-1 cells. Molecular markers are indicated.

**Fig 2:**
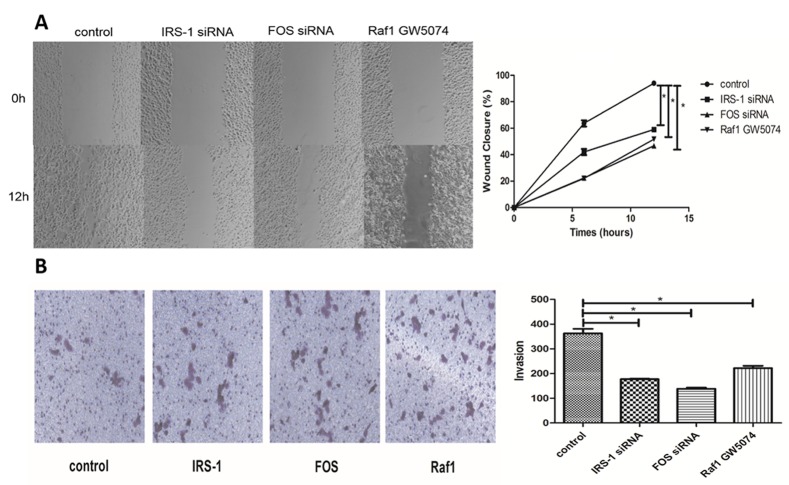
Differentially proteins play a major role in cell migration, and invasion of PC-1.0 Metastatic. (A) Effect of three proteins on the migration after using siRNA or antibody blocking in highly metastatic PC-1.0 cells. The cells were incubated for 24h. The percentage migration was calculated and graphed. * Compared with the PC-1.0, *P*-value <0.01. (n = 3) (B) Quantification of transwell assay for treated group and control group. Cells were counted in triplicate wells and in three identical experiments, * Compared with the PC-1.0, *P*-value <0.01. (n = 3)

To validate protein phosphorylation data, the expression levels of six randomly selected proteins from comparisons of PC-1.0 and PC-1 cells were re-examined by western blot (Fig 1). Western blots revealed that all six proteins showed increased phosphorylation in PC-1.0 cells consistent with our array data, thus confirming the latter’s reliability. To understand functional relationships of differential alteration in protein phosphorylation in highly metastatic PC-1.0 cells, invasion and migration assays were carried out. Western blot and real-time PCR analyses were performed to confirm the efficiency of siRNA or inhibitor down-regulation (As shown in S3 Table). A strong inhibition of migration was observed for FOS, IRS-1 and RAF1 using siRNA or antibody blocking (Fig 2A), and regarding invasion, FOS, IRS-1 and RAF1 also caused a significant reduction in the invasion ability (Fig 2B).

### Functional classification, network and pathway analysis

**Fig 3:**
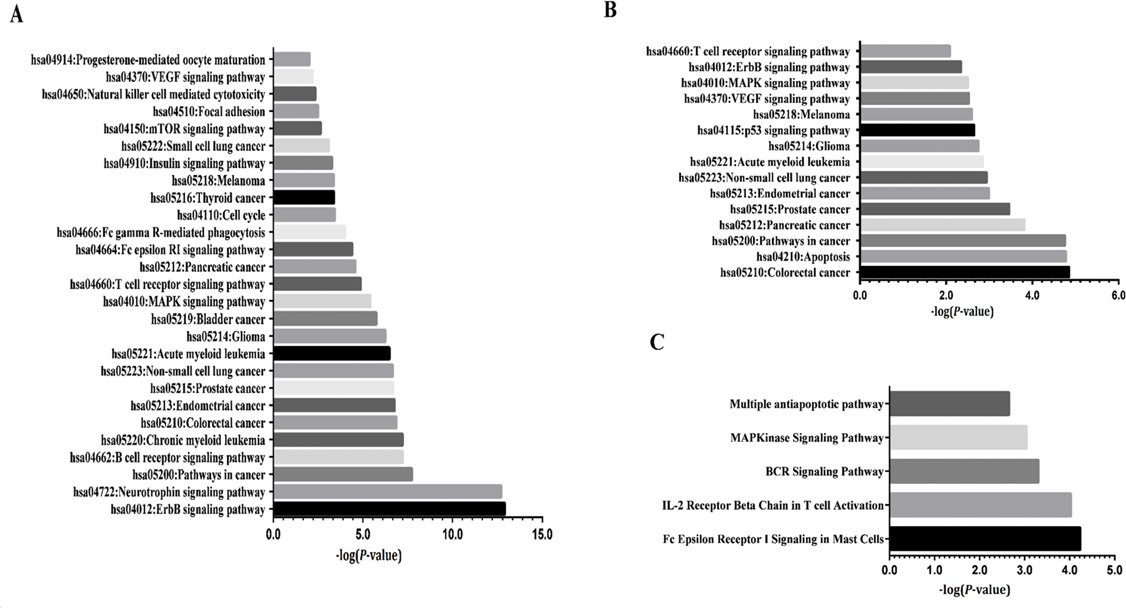
**KEGG and BioCarta pathway functional annotations of differentially expressed up-regulated (A, C) and down-regulated (B) proteins (*P*-values < 0.01, Benjamin < 0.05).** Enrichment scores corresponding to each pathway provided by the DAVID annotation tool are displayed as −log *P*-values. Down-regulated proteins that were BioCarta pathway functional annotations only were enriched in apoptotic signaling in response to DNA damage.

**Fig 4:**
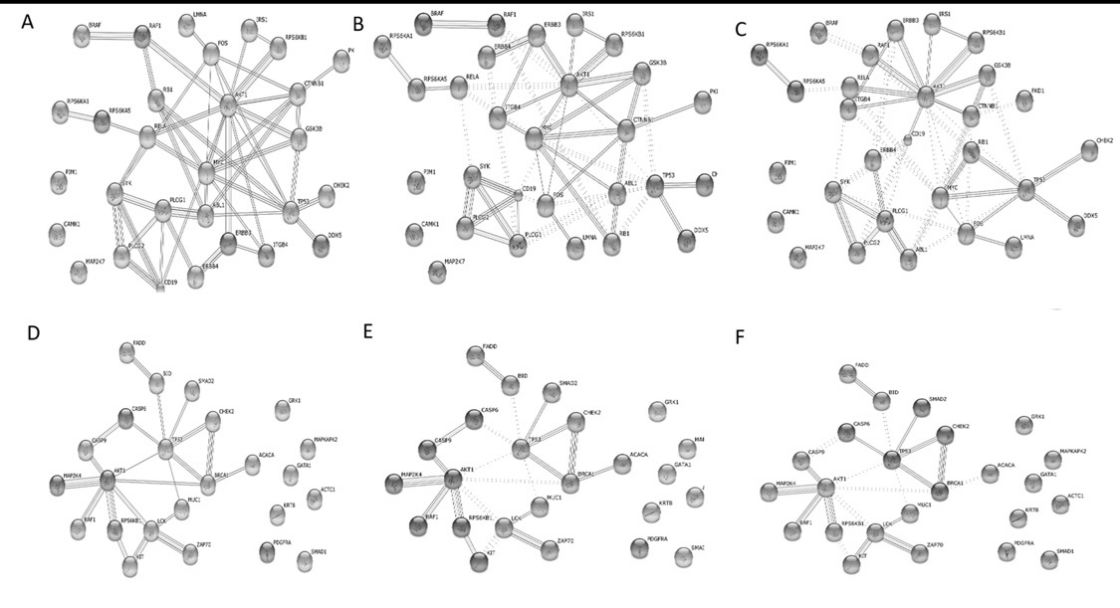
Protein-protein interaction network and signaling pathway analysis. (A) A PPI network, as shown in the interactive view, generated by a STRING database to reveal functional interactions between up-regulated proteins. Each node represents a protein, and each edge represents an interaction (*P*-values = 6.06E-12); (B) Up-regulated proteins’ MCL cluster algorithms derived using STRING. MCL = 2; (C) Up-regulated proteins’ K-Means cluster algorithms. K-Means = 4; approximate same consequences by two clusters; (D) A PPI network reveals functional interactions between down-regulated proteins (*P*-value = 1.47E-6); (E) Down-regulated proteins’MCL cluster algorithms. MCL = 2; (F) Down-regulated proteins’ K-Means cluster algorithms. K-Means = 4.

To understand the similar biological processes that correlate with invasion and metastasis of pancreatic cancer cells, we explored these again using the online functional annotation tool, DAVID, and including KEGG pathways analysis. GO term analysis showed that targets were enriched in many processes (Tables 3–5), including amino acid phosphorylation and post-translational protein modification. Pathway analyses revealed such up-regulated targets were enriched in 27 KEGG pathways (Fig 3A), which were essential for metabolism (insulin signaling pathway and cell cycle), immunity (T cell receptor signaling pathway) and development (ErbB signaling pathway). Proteins form a PPI network and are implicated in altering and controlling cellular invasion in diverse biological systems. The data are shown in Fig 3B and 3C. Fig 4A and 4D show the interaction network constructed in STRING after querying the 32 up-regulated and 25 down-regulated proteins found in PC-1.0 cells. In addition, when the confidence score was > 0.900, CAMK1-α, PIM-1and MKK7/MAP2K7 were no longer included in an up-regulated network, as well as MAPKAPK2, cytokeratin 8, GATA1, GRK1, ACC1, PDGFRA and Smad1 in a down-regulated network. Markov (MCL) and K-Means cluster algorithms were then used to further analyze PPI networks. By comparison, we obtained an approximate consequence of cluster where the MCL cutoff was 2 and the K-Mean cutoff was 4 in both up-regulated (Fig 4B and 4C) and down-regulated (Fig 4E and 4F) PPI networks. Proteins with fewer or no interactions was found more towards the periphery of the network. Further experimental investigation is required of these unrelated proteins in order to understand their real function in a PPI network.

**Table 3:** Gene Ontology analysis (top five): biological processes.

GO Term	Count	%	*P*-value	Benjamin	Genes
**GO:0006468 protein amino acid phosphorylation**	26	53.0	2.2E-22	3.4E-19	PKD1, Smad1, LCK, et al.
**GO:0016310 phosphorylation**	26	53.0	2.0E-20	1.6E-17	PKD1, Smad1, LCK, et al.
**GO:0006793 phosphorus metabolic process**	27	55.1	1.3E-19	5.1E-17	CDC25C, GRK1,MSK1, et al.
**GO:0006796 phosphate metabolic process**	27	55.1	1.3E-19	5.1E-17	Smad2, GSK3α-β, MKK7,et al.
**GO:0043687 post-translational protein modification**	28	57.1	1.7E-17	4.8E-15	CaMK1-α, BRCA1, P70S6K, et al.

**Table 4:** Gene Ontology analysis (top five): cellular components.

GO Term	Count	%	*P*-value	Benjamin	Genes
**GO:0005829 cytosol**	22	44.8	4.1E-12	7.4E-10	CASP1, Smad1, PKD1, et al.
**GO:0070013 nuclear lumen**	19	38.7	2.5E-9	2.3E-7	chk2, Smad1, P90RSK, et al.
**GO:0005667 intracellular organelle**	20	40.8	1.6E-8	9.3E-7	chk2, Smad1, P90RSK, et al.
**GO:0043233 organelle lumen**	20	40.8	2.2E-8	9.9E-7	chk2, Smad1, CASP9, et al.
**GO:0031974 nucleoplasm**	20	40.8	3.2E-8	1.1E-6	P53, AKT1, GATA1, et al.

**Table 5:** Gene Ontology analysis (top five): molecular functions.

GO Term	Count	%	*P*-value	Benjamin	Genes
**GO:0004672 protein kinase activity**	24	48.9	6.3E-22	1.4E-19	PKD1, chk2, Raf1, et al.
**GO:0016773 phosphotransferase activity**	24	48.9	4.4E-20	4.9E-18	PKD1, chk2, Raf1, et al.
**GO:0016301 kinase activity**	24	48.9	3.1E-19	2.3E-17	PKD1, ErbB4, Raf1, et al.
**GO:0016772 transferase activity**	24	48.9	1.5E-17	8.5E-16	LCK, GRK1, P90RSK, et al.
**GO:0005524 ATP binding**	27	55.1	1.4E-15	6.1E-14	P53, PKD1, P90RSK, et al.

**Fig 5:**
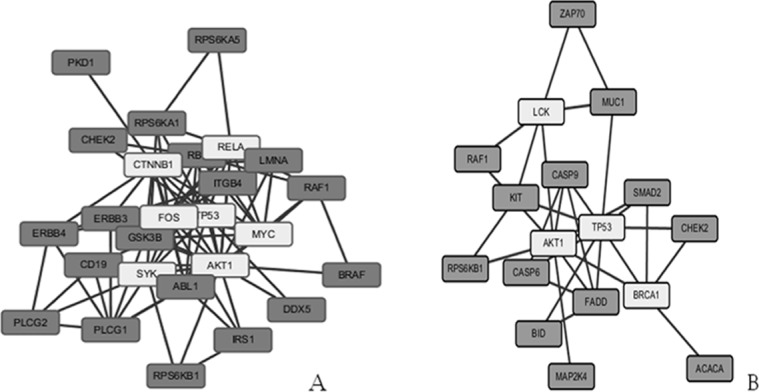
Key nodes of the PPI network visualized by Cytoscape software. All nodes that have a centrality value greater or equal to the threshold are highlighted with a light gray color in the network view. (A) up-regulated proteins; (B) down-regulated proteins.

Furthermore, CentiScaPe software, which calculate topological characteristics of each node, were used to gain an in-depth understanding of the biological characteristics of the PPI network, but not including unrelated proteins. The up-regulation PPI network consisted of 26 nodes with an average path length of 1.89. Node degree was 6.62±4.18, and betweenness was 22.23±35.72 (Table 6). With the same method, our down-regulation PPI network displayed 17 nodes with an average path length of 2.04. Node degree was 3.65±2.47, and betweenness was 16.59±29.58 (Table 7). Proteins with high value were more important in the network and this suggested a central role in maintaining functionality and the coherence of signaling mechanisms. In our up-regulated PPI network, hub proteins with high node degree and betweenness (> Means) were AKT1, CTNNB1, FOS, NFkB-p65, SYK, TP53 and MYC. On the other hand, the down-regulated network included BRCA1, LCK, AKT1, and TP53 (Fig 5). Notably, AKT1 and TP53 displayed the highest score for all computed centralities, suggesting its central regulatory role in up-regulated proteins and in a down-regulated network.

**Table 6:** Significantly enriched modular of up-regulated proteins using CentiScaPe software.

Name	Degree	Betweenness
**RELA**	10	48.98413
**RPS6KA1**	5	11.27143
**AKT1**	18	166.123
**ITGB4**	3	0.4
**DDX5**	2	0
**RPS6KB1**	3	0.916667
**LMNA**	5	0.666667
**TP53**	14	77.22302
**RPS6KA5**	2	0
**GSK3B**	6	3.383333
**ABL1**	8	14.81111
**RB1**	8	17.23889
**CTNNB1**	11	65.0381
**FOS**	13	51.07619
**ERBB3**	7	12.30476
**MYC**	11	24.11349
**CD19**	6	9.566667
**SYK**	9	35.43889
**PLCG1**	8	18.93571
**PKD1**	1	0
**CHEK2**	2	0
**ERBB4**	5	9.072222
**PLCG2**	4	1.916667
**RAF1**	5	6.152381
**BRAF**	2	0
**IRS1**	4	3.366667
**RELA**	10	48.98413
**RPS6KA1**	5	11.27143

**Table 7:** Significantly enriched modular of down-regulated proteins using CentiScaPe software.

Name	Degree	Betweenness
**BRCA1**	5	35.16666667
**TP53**	10	93.2
**CASP9**	4	2.9
**FADD**	5	9.066666667
**ACACA**	1	0
**AKT1**	9	93.03333333
**MAP2K4**	1	0
**BID**	2	0
**RPS6KB1**	2	1.333333333
**SMAD2**	3	0
**LCK**	5	24.33333333
**CHEK2**	2	0
**CASP6**	3	0
**ZAP70**	2	0
**MUC1**	3	15.06666667
**KIT**	3	7.9
**RAF1**	2	0

**Fig 6:**
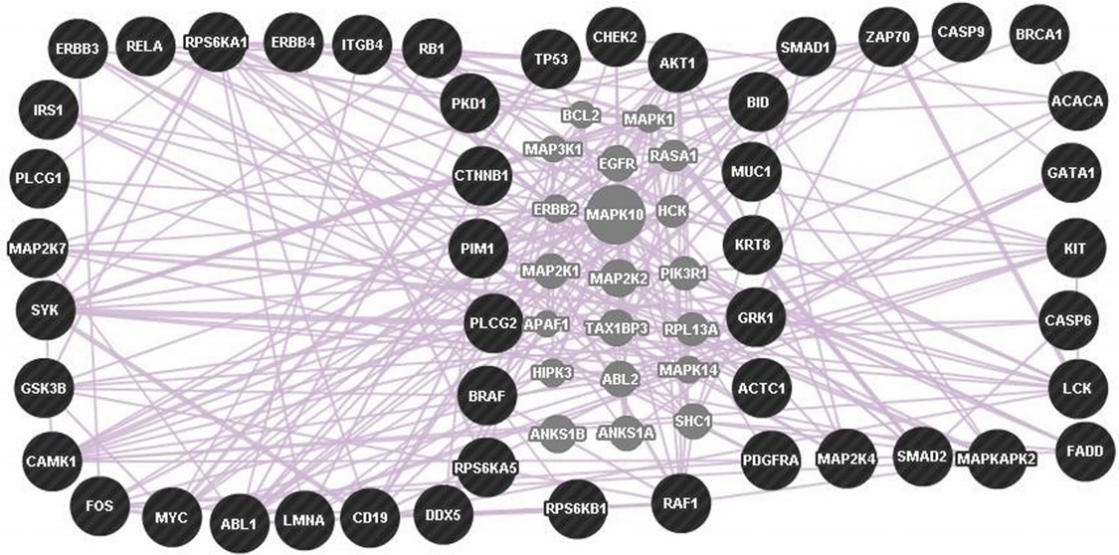
Co-expression gene network. A network of differential genes was generated using GeneMANIA. The left network represents up-regulated genes and the right network represents down-regulated genes. In addition, five genes exist in both the up- and down-regulated networks (TP53, CHEK2, AKT1, RPS6KB1, RAF1). Query genes are indicated by black dots and co-expression is indicated by pink lines. Gray nodes indicate predicted genes.

Next, differentially expressed phosphoproteins were uploaded onto a GeneMANIA tool to predict a co-expression network. As a result, 20 genes were added to the network (Fig 6, Table 8). Such a co-expression network helps to explain distinct processes found in pancreatic cancer cell lines. In general, such topological properties of the network may improve our understanding of key genes associated with invasion.

**Table 8:** List of co-expressed phosphoproteomes with differential expression and sources of information on gene expression patterns using the GeneMANIA tool. (Weight ≥ 0.04).

Gene 1	Gene 2	Weight	Network group	Source
**SYK**	**PLCG2**	0.0914	Co-expression	Alizadeh-Staudt-2000; Bild-Nevins-2006 B; Burington-Shaughnessy-2008; Cheok-Evans-2003; Ramaswamy-Golub-2001; Salaverria-Siebert-2011
**SYK**	**CD19**	0.0774	Co-expression	Alizadeh-Staudt-2000; Bild-Nevins-2006 B; Cheok-Evans-2003; Innocenti-Brown-2011; Kang-Willman-2010; Salaverria-Siebert-2011
**LCK**	**ZAP70**	0.0673	Co-expression	Arijs-Rutgeerts-2009; Bild-Nevins-2006 B; Cheok-Evans-2003; Innocenti-Brown-2011; Kang-Willman-2010; Ramaswamy-Golub-2001
**MAPK10**	**CaMK1**	0.0589	Co-expression	Alizadeh-Staudt-2000; Burington-Shaughnessy-2008; Salaverria-Siebert-2011
**RPS6KB1**	**DDX5**	0.0538	Co-expression	Bild-Nevins-2006 B; Gysin-McMahon-2012; Wang-Maris-2006
**MAPK1**	**HIPK3**	0.0489	Co-expression	Cheok-Evans-2003; Gysin-McMahon-2012; Wang-Maris-2006
**PLCG2**	**CD19**	0.0483	Co-expression	Alizadeh-Staudt-2000; Arijs-Rutgeerts-2009; Bild-Nevins-2006 B; Cheok-Evans-2003; Salaverria-Siebert-2011
**MAP2K7**	**GRK1**	0.0471	Co-expression	Bild-Nevins-2006 B; Salaverria-Siebert-2011; Wu-Garvey-2007
**MAPK14**	**MAPK1**	0.0467	Co-expression	Bild-Nevins-2006 B; Burington-Shaughnessy-2008; Salaverria-Siebert-2011
**MYC**	**FOS**	0.0463	Co-expression	Innocenti-Brown-2011; Smirnov-Cheung-2009; Wang-Maris-2006
**HCK**	**RPS6KA1**	0.0436	Co-expression	Boldrick-Relman-2002; Innocenti-Brown-2011; Kang-Willman-2010; Salaverria-Siebert-2011
**MAPK1**	**RB1**	0.0414	Co-expression	Bild-Nevins-2006 B; Kang-Willman-2010
**RASA1**	**SMAD2**	0.0404	Co-expression	Arijs-Rutgeerts-2009; Burington-Shaughnessy-2008

## Discussion

In recent years, many researchers have used endoscopic ultrasound-guided fine needle aspiration (EUS-FNA) as a preoperative diagnostic method to reduce unnecessary surgical procedures in an effort to achieve higher survival rates [14,15]; however, results have been unsatisfactory. How to reduce invasion and metastasis of pancreatic cancer is still an intense area of research and ongoing problem. Phosphorylation is the most important and is involved in many cellular processes (e.g. proliferation, differentiation, signal transduction, metabolism, apoptosis, cellular-signaling, transcriptional and translational regulation) [16–23].

As described in our previous study, we have established two pancreatic cancer cell lines from an experimental pancreatic cancer model [5,6]. Non-dissociated cells (PC-1) and dissociated cells (PC-1.0) show high histological similarities but differ in their invasive and metastatic capabilities. We rationalized that using such cell lines would minimize the influence of using cells from different tissue origins. Therefore, the analysis of differentially expressed phosphoproteomes in two cell lines (PC-1.0 and PC-1) is particularly important for the study of pancreatic cancer invasion and metastasis mechanisms.

In order to further understand different biological functions produced by different phosphorylation modifications of the same protein in the two cell lines (PC-1.0 and PC-1), a high-throughput phosphorylation array technique was used in our analyses. The array detected phosphorylation levels of proteins on specific amino acid residues (e.g. Ser, Thr, Tyr), and provided accurate and efficient phosphoproteome profiling of each pancreatic cancer cell line. After data mining, we have observed that of the 57 signature differential expression proteins, six genes encoded 12 proteins, with phosphorylation occurring at different sites (i.e. NFkB-p65, P70S6K, Smad2, Chk2, p53 and AKT1). The site-specific phosphorylation of proteins normally results in different biological functions [24]. But the roles of multiple phosphorylation sites in a single protein molecule have not been clearly elucidated. Therefore, Guo et al. used nanofluidic proteomic immunoassay (NIA) to analyze and characterize protein phosphorylation of multiple different sites [25]. Compared to the Guo et al. study, our results indicate that phosphorylation at Thr72 and Ser124 of AKT1 have a more important role and may be closely related to pancreatic cancer invasion and metastasis.

Generally speaking, chemokines are small proteins that play an important role in controlling the migration of diverse cells [26]. Our results show that 10 proteins that we identified could be classed as chemokines (i.e. ITGB4, PLCG2, IRS-1, FOS, CTNNB1, PLCG1, CD227, PKD1, P53 and ACTC1), according to http://www.phosphosite.org [27]. Other invasion-related protein types were transcription factors (MYC, Rb, FOS, CTNNB1, NFkB-p65, P53, BRCA1, GATA1, Smad1 and Smad2) and cytoskeletal proteins (lamin A, cytokeratin 8 and ACTC1).

Through KEGG pathway analyses, we found that these differential expression proteins existed in two main signaling pathways (ErbB and neurotrophin signaling pathways as shown in Fig 2A). Among the altered pathway, we found PI3K and MAPK as main actors in the process of cell motility. In our previous studies, we found that activation of the EGFR mediated MEK/ERK signaling pathway induced cell invasion in the PC-1 cells, conversely, EGFR inhibitor AG1478 could inhibit above pathway and reduce cell invasion in the PC-1.0 cells [28]. In this study, we detected hyperphosphorylation of ErbB3, ErbB4, Raf1, P90RSK, MSK1, MYC and FOS in the PC-1.0 cells compared with PC-1 cells, but changes in Grb2, MEK, ERK and Elk1 were not observed. Furthermore, we employed Raf1 inhibitor and FOS siRNA to study the relationship between protein and invasion. The results revealed that the knockdown of FOS or Raf1 significantly inhibited the migration and invasion of the PC-1.0 cells. Our results, Grb2/MEK/ERK signaling were not observed, maybe result from methodologic limitations. Our data showed that P90RSK was hyperphosphorylated, however, Kang et al. reported that P90RSK2 promoted invasion and metastasis of human head and neck squamous cell carcinoma (HNSCC) [29]. P90RSK may be involved in the regulation of pancreatic cancer cell invasion and metastasis.

The other signaling pathway (PI3K/AKT) usually involves in cell proliferation and cell cycle [30]. In our data, we tested several upstream regulators of PI3K/AKT such as VEGFR1/2/3, ErbB2/3/4, PDGFRα, MET, EGFR, FLT3, KIT, and IGF-1R. Among these proteins, ErbB3 and ErbB4 are highly phosphorylated, in contrast, PDGFRα and KIT are significantly decreased. Meanwhile, we observed that IRS-1 phosphorylation at Ser 636 was markedly increased, but IGF-1R was not changed. Notably, IRS-1 phosphorylation can be negatively regulated by activation of P70S6K in the PI3K/AKT/mTOR signaling pathway [31]. In this study, we found an increase in P70S6K phosphorylation at Ser371 and Thr 421, and decrease at Ser 418 phosphorylation. Therefore, our studies suggested that PDGFRα and KIT downregulation in pancreatic cancer leads to decreased p70S6K phosphorylation at Ser 418, and increased IRS-1 phosphorylation at Ser 636 via increased PI3K/AKT mTOR/P70S6K signaling. Hyperphosphorylation of IRS-1 subsequently mediated activation of PI3K/AKT pathway. The finding could indicate the mechanism of resistance in pancreatic cancer, as a result, combination clinical trials in pancreatic cancer should be considered.

In summary, identifying differentially expressed phosphoproteomes, as revealed in our study, will point to the mechanisms underlying the invasion and metastasis of pancreatic cancer. In future, we need pay more attention to identifying the activated phosphoproteome, particularly specific amino acid residues. Taken together, the identification of abnormal protein phosphorylation, which is related to invasion and metastasis, may allow us to identify new biomarkers for the early detection of pancreatic cancer.

## Supporting Information

S1 Table Primer sequences and inhibitor used in the assay. Real-time PCR was performed to analyze the level of FOS or IRS-1 mRNA in the knockdown cell lines. The level of Raf1 knockdown was confirmed using Western blot.
(DOCX)

S2 Table The Phospho Explorer Antibody Array was scanned on a GenePix 4000 scanner. (DOCX)

S3 Table The expression of all proteins of the Phospho Explorer Antibody Array were presented. The coordinates and proteins were indicated in the table.
(XLSX)
