# Supplementary material for: Phosphoproteome Analysis of Invasion and Metastasis-Related Factors in Pancreatic Cancer Cells
Source: PLoS One. 2016 Mar 25;11(3):e0152280. doi: 10.1371/journal.pone.0152280 (PMC4807880; doi:10.1371/journal.pone.0152280)
Supplement: S1 Table — Real-time PCR was performed to analyze the level of FOS or IRS-1 mRNA in the knockdown cell lines. The level of Raf1 knockdown was confirmed using Western blot. (DOCX) [file pone.0152280.s001.docx]

FOS: F Primer: TCTTACTACCACTCACCCGCAGAC

R Primer: GGAATGAAGTTGGCACTGGAGAC

IRS-1: F Primer: ACAGGGTGGGCCAAATTAAAC

R Primer: ACCATGCATTGGTCTTTGTGTA

Raf1: GW5074 is the Raf1 inhibitor.


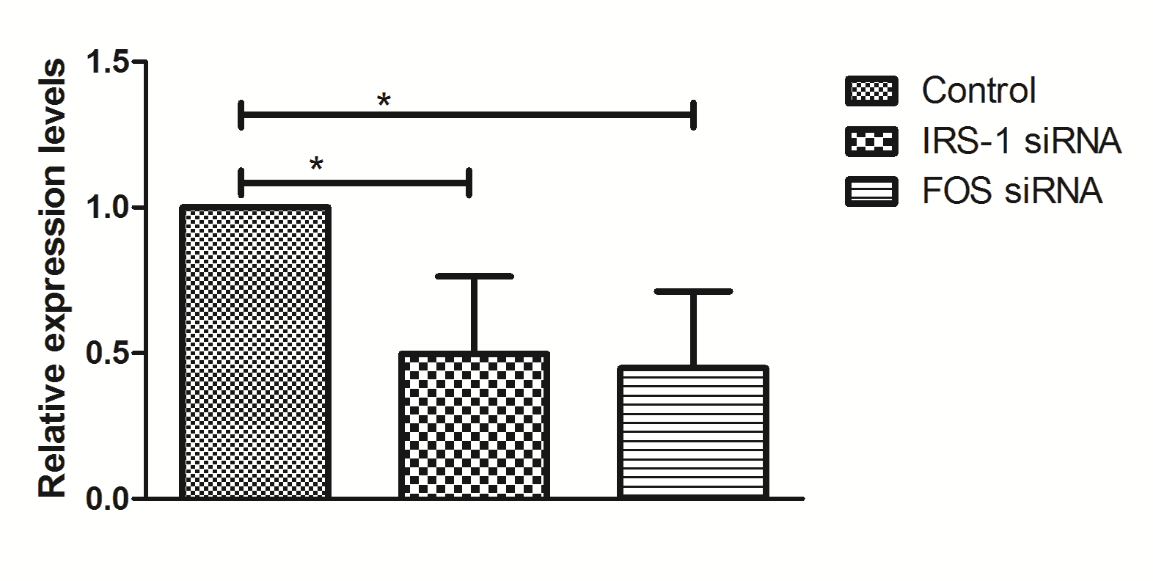
*, *P* < 0.01
