# Supplementary material for: Phosphoproteome Analysis of Invasion and Metastasis-Related Factors in Pancreatic Cancer Cells
Source: PLoS One. 2016 Mar 25;11(3):e0152280. doi: 10.1371/journal.pone.0152280 (PMC4807880; doi:10.1371/journal.pone.0152280)
Supplement: S2 Table — (DOCX) [file pone.0152280.s002.docx]

Phospho Explorer Antibody Array is a high-throughput antibody array, contained 1318 antibodies. Each array contained 16 blocks, the pre-coated antibodies on the left 8 blocks was the same as the right. GAPDH and beta-actin served as positive controls

The fluorescence signal (I) of each antibody was obtained from the fluorescence intensity of this antibody spot. We normalized the data sets against the median fluorescence signal means to minimize the effect of both sides signal intensity. A ratio computation was used to measure the extent of protein phosphorylation. A phosphorylation ratio change was computed based on the following equation where expression of phosphorylated proteins was normalized to corresponding unphosphorylated protein expression in both experimental (PC-1.0) and reference data (PC-1). Phosphorylated proteins were considered to be differentially expressed when an increase (≥ 2.0) or decrease (≤ 0.5) occurred in the ratio of expression levels compared against control group.
